# Efficacy and safety of recanalization therapy for acute ischemic stroke with COVID-19: A systematic review and meta-analysis

**DOI:** 10.3389/fneur.2022.984135

**Published:** 2022-08-30

**Authors:** Zilan Wang, Haiying Teng, Xiaoxiao Wu, Xingyu Yang, Youjia Qiu, Huiru Chen, Zhouqing Chen, Zhong Wang, Gang Chen

**Affiliations:** ^1^Department of Neurosurgery and Brain and Nerve Research Laboratory, The First Affiliated Hospital of Soochow University, Suzhou, China; ^2^Suzhou Medical College of Soochow University, Suzhou, China; ^3^Department of Neurology, The First Affiliated Hospital of Soochow University, Suzhou, China

**Keywords:** acute ischemic stroke, COVID-19, intravenous thrombolysis, mechanical thrombectomy, meta-analysis, recanalization therapy

## Abstract

**Background:**

The novel coronavirus disease 2019 (COVID-19) has rapidly spread worldwide and created a tremendous threat to global health. Growing evidence suggests that patients with COVID-19 have more severe acute ischemic stroke (AIS). However, the overall efficacy and safety of recanalization therapy for AIS patients infected by the SARS-CoV-2 virus is unknown.

**Methods:**

The PRISMA guideline 2020 was followed. Two independent investigators systematically searched databases and ClinicalTrials.gov to identify relevant studies published up to 31 March 2022. AIS patients who received any recanalization treatments were categorized into those with COVID-19 and those without COVID-19. The main efficacy outcomes were patients' functional independence on discharge and successful recanalization, and the safety outcomes were in-hospital mortality and symptomatic intracranial hemorrhage. Subgroup analyses were implemented to assess the influence of admission National Institutes of Health Stroke Scale and different recanalization treatments on the outcomes. STATA software 12.0 was used for the statistical analysis.

**Results:**

This systematic review and meta-analysis identified 10 studies with 7,042 patients, including 596 COVID-19 positive patients and 6,446 COVID-19 negative patients. Of the total patients, 2,414 received intravenous thrombolysis while 4,628 underwent endovascular thrombectomy. COVID-19 positive patients had significantly lower rates of functional independence at discharge [odds ratio (OR) 0.30, 95% confidence interval (CI) 0.15 to 0.59, *P* = 0.001], lower rates of successful recanalization (OR 0.40, 95% CI 0.24 to 0.68, *P* = 0.001), longer length of hospital stay (weighted mean difference 5.09, 95% CI 1.25 to 8.94, *P* = 0.009) and higher mortality rates (OR 3.38, 95% CI 2.43 to 4.70, *P* < 0.0001). Patients with COVID-19 had a higher risk of symptomatic intracranial hemorrhage than the control group, although the difference did not reach statistical significance (OR 2.34, 95% CI 0.99 to 5.54, *P* = 0.053).

**Conclusions:**

Compared with COVID-19 negative AIS patients who received recanalization treatments, COVID-19 positive patients turned out to have poorer outcomes. Particular attention needs to be paid to the treatments for these COVID-19 patients to decrease mortality and morbidity. Long-term follow-up is necessary to evaluate the recanalization treatments for AIS patients with COVID-19.

**Systematic review registration:**

https://inplasy.com/inplasy-2022-4-0022/, identifier: INPLASY202240022.

## Introduction

Since the first case of severe acute respiratory syndrome coronavirus 2 (SARS-CoV-2) infection was reported in late December 2019, the world has witnessed an overwhelming global coronavirus infectious disease 2019 (COVID-19) pandemic. The COVID-19 pandemic has led to millions of confirmed cases and deaths now (1). Acute cerebrovascular diseases are frequently reported in patients with COVID-19 infection, and the most common manifestation is acute ischemic stroke (AIS) (2). An observational study reported an AIS prevalence of 4.6% in patients with COVID-19 infection (3), which is higher than that reported in patients without COVID-19 (4), or patients with influenza (5) or SARS (6). What's worse, AIS has been reported to be one of the most severe complications of COVID-19 infection. There are accumulating reports suggesting that COVID-19 patients with AIS present with worse functional outcomes and higher mortality than COVID-19 patients without AIS (7–10). Additional studies revealed that the potential stroke mechanisms in COVID-19 are hypercoagulopathy, angiotensin-converting enzyme 2 inhibition, and cardioembolism (11). A multicenter retrospective study of South India suggested that hypertension and atrial fibrillation were more common in the COVID-19 related stroke group than in historical controls (9). In addition, during the COVID-19 infection, hypercoagulability arises as a “sepsis-induced like coagulopathy” and may predispose patients to AIS (12). Nannoni et al. (2) suggested that hypercoagulation could lead to an increased risk of cerebral thrombosis and/or thromboembolism, which may be the reason why AIS is common in patients with COVID-19.

Intravenous thrombolysis (IVT) was the main systemic reperfusion therapy for AIS and was usually performed within 4.5 h of symptom onset (13, 14), while effective endovascular therapy showed improved outcomes and expanded the window period of therapy in AIS (15). However, the pandemic of COVID-19 has notable impacts on the treatments and management of AIS patients, and recanalization therapy during the pandemic could be challenging. A study from China reported that both prehospital (onset-to-door time) and posthospital (door-to-needle time) delay were prolonged remarkably, and the proportion of patients with AIS who received IVT treatment decreased significantly (16). Pooled analysis of July et al. (17) showed a significant reduction in mechanical thrombectomy performed during the pandemic than during the pre-pandemic period. Untimely recognition of stroke due to quarantine, delayed patient arrivals, COVID-19 screening before admission, and preparation of protective equipment for stroke team members, may lead to the missing of the therapeutic window (18). While in some researches, the recanalization therapies were not delayed during the COVID-19 pandemic (9, 19, 20).

Because of the rapidly increasing number of AIS complications combined with COVID-19 infections, it is of vital importance to have an in-depth understanding of the impact of COVID-19 on recanalization therapy for these patients. However, the published literature was limited to case reports, case series, and observational studies. The overall effect of COVID-19 on the outcomes of recanalization therapy for AIS patients has not been adequately assessed. Thus, we performed a meta-analysis to evaluate the efficacy and safety of recanalization therapy for COVID-19 patients who suffered from AIS.

## Methods

### Data availability

The authors declare that all supporting data are available within the article and in the Supplemental material.

### Study protocol

We registered our study on INPLASY website (Register number INPLASY202240022) and followed the Preferred Reporting Items for Systematic Reviews and Meta-Analyses (PRISMA) 2020 statement (21).

### Eligibility criteria

We set the inclusion criteria as follows: (1) study type: retrospective, prospective cohort study, or randomized controlled trial (RCT) study design; (2) language: published in English; (3) participants: AIS patients (≥18 years) received any recanalization treatments, with or without COVID-19 infection; COVID-19 infection was laboratory-confirmed by PCR or antigen test. (4) interventions: patients were categorized into those with COVID-19 versus those without COVID-19, and treated with IVT, intraarterial thrombolysis (IAT), endovascular thrombectomy (EVT), or a combination of these recanalization interventions; (5) outcomes: including efficacy and safety outcomes. The primary efficacy outcome was functional independence on discharge (modified Rankin Scale, mRS 0–2). The second efficacy outcome was successful recanalization indicated by Thrombolysis in Cerebral Infarction (TICI) or modified TICI (mTICI) scores ≥ 2 b/3. Other efficacy outcomes include the length of hospital stay (days), time (min) from stroke onset to treatment (onset-to-needle in those who received IVT or combined therapy; onset-to-groin puncture in those who received EVT or combined therapy), and time (min) from door to treatment (door-to-needle in those who received IVT or combined therapy; door-to-groin puncture in those who received EVT or combined therapy). The safety outcomes were in-hospital mortality and symptomatic intracranial hemorrhage (sICH), defined as worsening of 4 or more points in NIHSS attributed to hemorrhagic transformation. Included studies were not requested to supply all the outcomes mentioned above.

We set the exclusion criteria as follows: (1) review, commentary, letter, case reports, case-series, or observational studies without a control group. (2) Not all the AIS patients included in the study received recanalization interventions. (3) Suspected/ probable cases of SARS-CoV-2 infection.

### Search strategy

Two independent investigators (ZiW and HT) systematically searched MEDLINE, EMBASE, the Cochrane Central Register of Controlled Trials (CENTRAL), and ClinicalTrials.gov to identify relevant studies published up to 31 March 2022. We searched Medical Subject Headings (MeSH) terms and keywords (in the title/abstract) in multiple combinations, including *COVID-19, SARS-CoV-2, 2019-nCoV, stroke, cerebrovascular disease, cerebral infarction, thrombolysis, thrombectomy, thrombolytic, revascularization, recanalization*. The detailed search strategy was described in Supplementary Table S1. Additionally, relevant systematic reviews and meta-analyses were also screened independently and manually to ensure a more comprehensive search.

### Study selection and data collection

According to the eligibility criteria mentioned above, two investigators (XW and XY) independently evaluated all records retrieved from the databases and relevant systematic reviews or meta-analyses. Disagreements were resolved by discussion or by another independent investigator (ZC). After selection and evaluation, data from the included studies were extracted, including study authors and year, publications, study design, basic information of the patients, inclusion and exclusion criteria, and outcome events.

### Risk of bias

The risk of bias for each included study was assessed using the Methodological Index for Non-randomized Studies (MINORS) for all included studies (22). MINORS contains 12 items relating to potential areas of bias. Each item receives a score from 0 to 2, resulting in overall scores ranging from 0 to 24. Two investigators performed the assessment independently (ZiW and HT). Disagreements were solved between the two investigators by consensus or by another independent investigator (ZC).

### Statistical analysis

STATA software 12.0 (STATA Corp., College Station, Texas, USA) was used for the statistical analysis. The Meta-Analyses were based on a random-effects model. Weighted mean difference (WMD) and 95% confidence interval (CI) were calculated for the continuous outcomes. Odds ratio (OR) and 95% CI values were calculated for the dichotomous outcomes. A funnel plot was used to investigate possible publication bias, true heterogeneity and other methodological irregularities. Cochrane's Q test and *I*^2^ were used for calculating outcome heterogeneity. Sensitivity analysis was also performed to explore the stability of the consolidated results. For all the analyses, two-tailed tests were performed, and *P* < 0.05 was considered to be statistically significant.

## Results

### Search results and study characteristics

**Figure 1:**
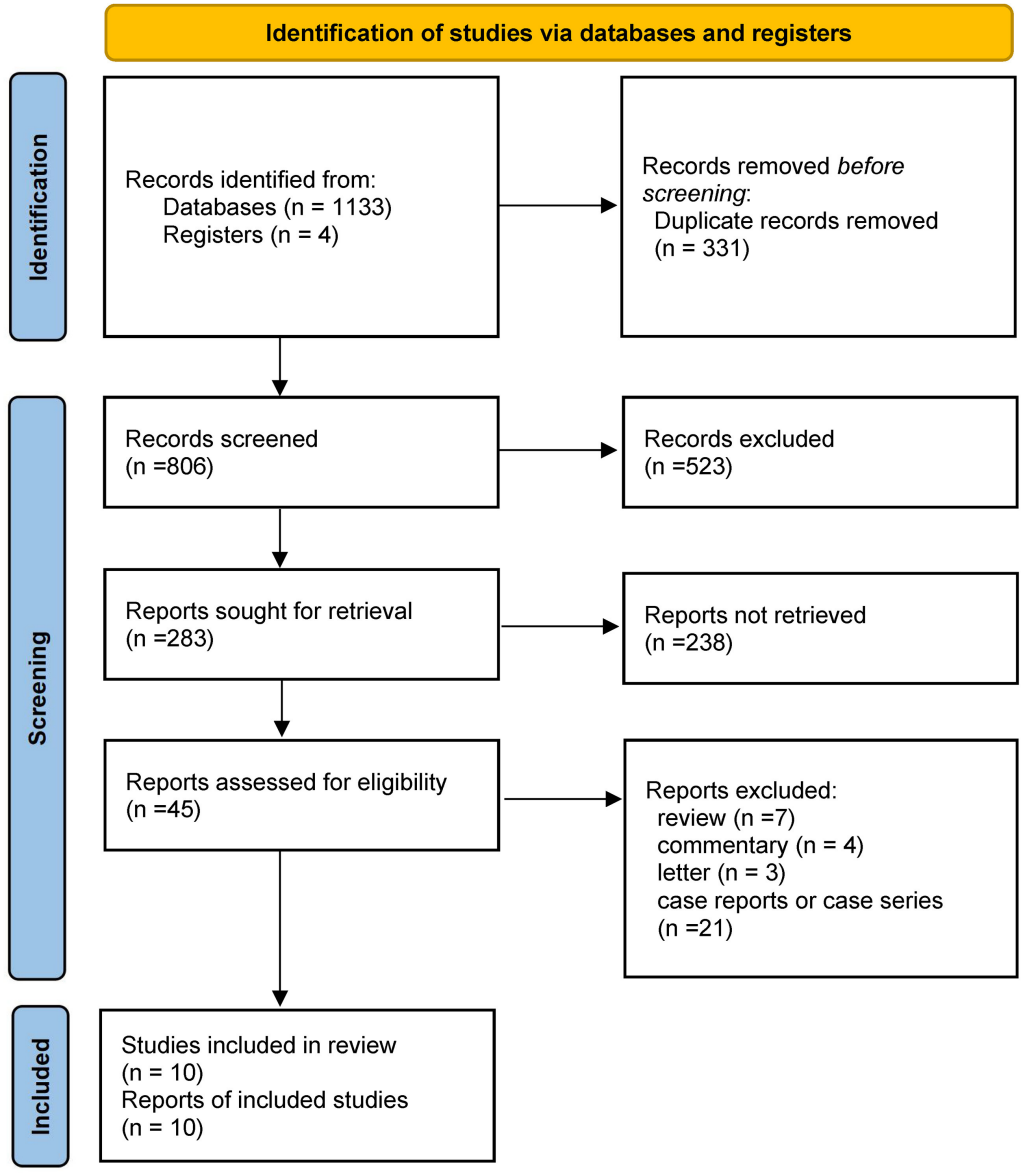
PRISMA flow diagram of study selection.

MEDLINE, EMBASE, CENTRAL, and ClinicalTrials.gov provided 1,137 titles and abstracts for review. Of these, a total of 331 articles were excluded due to duplication. After screening, 45 full articles were further assessed for eligibility. Eventually, ten studies (19, 20, 23–30) containing 7,042 patients (596 in the COVID-19 positive group and 6,446 in the COVID-19 negative group) were selected for qualitative synthesis (Figure 1). Five were retrospectively designed and five were prospectively designed. Six studies mainly focused on EVT treatment, two on IVT treatment, and two have data on both EVT and IVT treatments. The main characteristics of the included studies were listed in Table 1. Other details of the studies were shown in the supplementary materials (Supplementary Table S2).

**Table 1:** Characteristics of the included studies.

**Autho (year)**	**Countries**	**Centers**	**Publications**	**Study design**	**Group (No. of participates)**		**Male (N, %)**		**Age (year)**		**Baseline NIHSS**	
					**COVID-19**	**non-**	**COVID-19**	**non-**	**COVID-19**	**non-**	**COVID-19**	**non-**
						**COVID-19**		**COVID-19**		**COVID-19**		**COVID-19**
Al Kasab 2020 (19)	USA, Germany, Switzerland, Portugal, and Uruguay	28	BMJ The Pandemic and Neurointervention	Prospective	13	445	8 (61.5%)	240 (53.9)	58 (50–71)	72 (60–80)	19 (16–24)	15 (10–20)
de Havenon 2020 (23)	United States	Vizient Clinical Data Base	BMJ The Pandemic and Neurointervention	Retrospective	104	3061	71 (68.3%)	1571 (51.3%)	NA	NA	NA	NA
Escalard 2020 (20)	France	1	Stroke	Prospective	10	27	8 (80%)	13 (48%)	59.5 (54–71.5)	72 (60-81.5)	22 (19–25.7)	16 (12.5–19.5)
Pezzini 2021 (25)	Northern Italy	10	Journal of Neurology	Prospective	34	262	24 (70.6%)	130 (49.6%)	76 (63–82.25)	74 (61–80)	12 (7–20.25)	10 (6–16)
Sasanejad 2021 (27)	Iran, Greece Germany	9	Journal of Stroke and Cerebrovascular Diseases	Prospective	101	444	60 (59.41%)	243 (54.85%)	68.1 ± 13.3	68.34 ± 14.5	13 (9–19)	11 (7–17)
Genchi 2022 (24)	Italy and Switzerland	2	Acta Neuropathologica Communications	Prospective	7	23	4 (57.1%)	10 (43.4%)	70.9 ± 12.4	74.7 ± 9.6	24 (20–26)	16 (9–22)
Qureshi 2022 (26)	United States	62	Journal of Stroke and Cerebrovascular Diseases,	Retrospective	96	1588	63 (65.6%)	799 (50.3%)	69.8 ± 13.5	70.5 ± 13.7	NA	NA
Sobolewski 2022 (29)	Poland	4	Acta Neurol Scand	Retrospective	22	48	15 (65.5%)	21 (42.0%)	74.5 ± 7.9	72.9 ± 12.8	11 (3–20)	6.5 (2–25)
Jabbour 2022 (30)	NA	50	Neurosurgery	Retrospective	194	381	NA	NA	62.5	71.2	NA	NA
Sawczyńska 2022 (28)	Poland	1	Neurologia i Neurochirurgia Polska	Retrospective	15	167	7 (46.7%)	84 (50.3%)	70	70	13.3 ± 6.6	15.5 ± 8

NIHSS, National Institutes of Health Stroke Scale.

### Efficacy outcome

**Figure 2:**
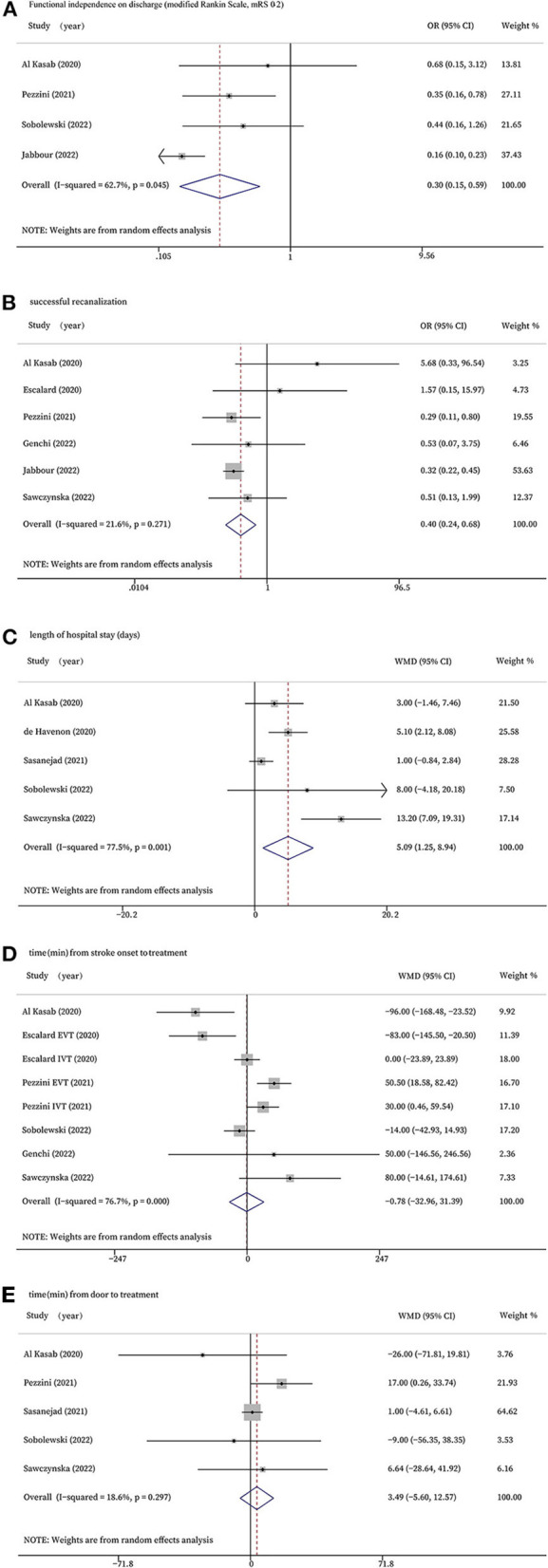
Forest plots for efficacy outcomes. **(A)**: Functional independence on discharge (modified Rankin Scale, 0-2); **(B)**: successful recanalization; **(C)**: length of hospital stay (days); **(D)**: time (min) from stroke onset to treatment; **(E)**: time (min) from door to treatment.

Four studies reported functional independence on discharge (mRS 0–2). According to the meta-analysis, the COVID-19 positive group showed significantly lower rates of functional independence than the COVID-19 negative group at discharge (OR 0.30, 95% CI 0.15 to 0.59, *P* = 0.001; Figure 2A). However, statistically significant evidence of heterogeneity across the five studies was found (*I*^2^ = 62.7%, *P* = 0.045). A sensitivity analysis was performed to detect the source of this statistical heterogeneity, demonstrating that the statistics were robust (Supplementary Figure S1).

Six studies that treated patients with EVT evaluated the rates of successful recanalization. The forest plot showed COVID-19 positive patients had significantly lower rates of successful recanalization (OR 0.40, 95% CI 0.24 to 0.68, *P* = 0.001, *I*^2^ = 21.6%; Figure 2B).

Five of the studies investigated the length of hospital stay and found stroke patients with COVID-19 infection stayed in the hospital significantly longer than those without COVID-19 infection (WMD 5.09, 95% CI 1.25 to 8.94, *P* = 0.009, *I*^2^ = 77.5%; Figure 2C). The sensitivity analysis validated the robustness of the results (Supplementary Figure S2).

Six studies reported time from stroke onset to treatment, and two of the studies had data of onset-to-needle in those who received IVT and onset-to-groin puncture in those who received EVT, respectively. There was no significant difference between the two groups in terms of time from stroke onset to treatment (WMD −0.78, 95% CI −32.96 to 31.39, *P* = 0.962, *I*^2^ = 76.7%; Figure 2D). The sensitivity analysis validated the robustness of the results (Supplementary Figure S3).

Five studies reported time from door to treatment, but no significant difference was found (WMD 3.49, 95% CI −5.60 to 12.57, *P* = 0.452, *I*^2^ = 18.6%; Figure 2E).

### Safety outcome

**Figure 3:**
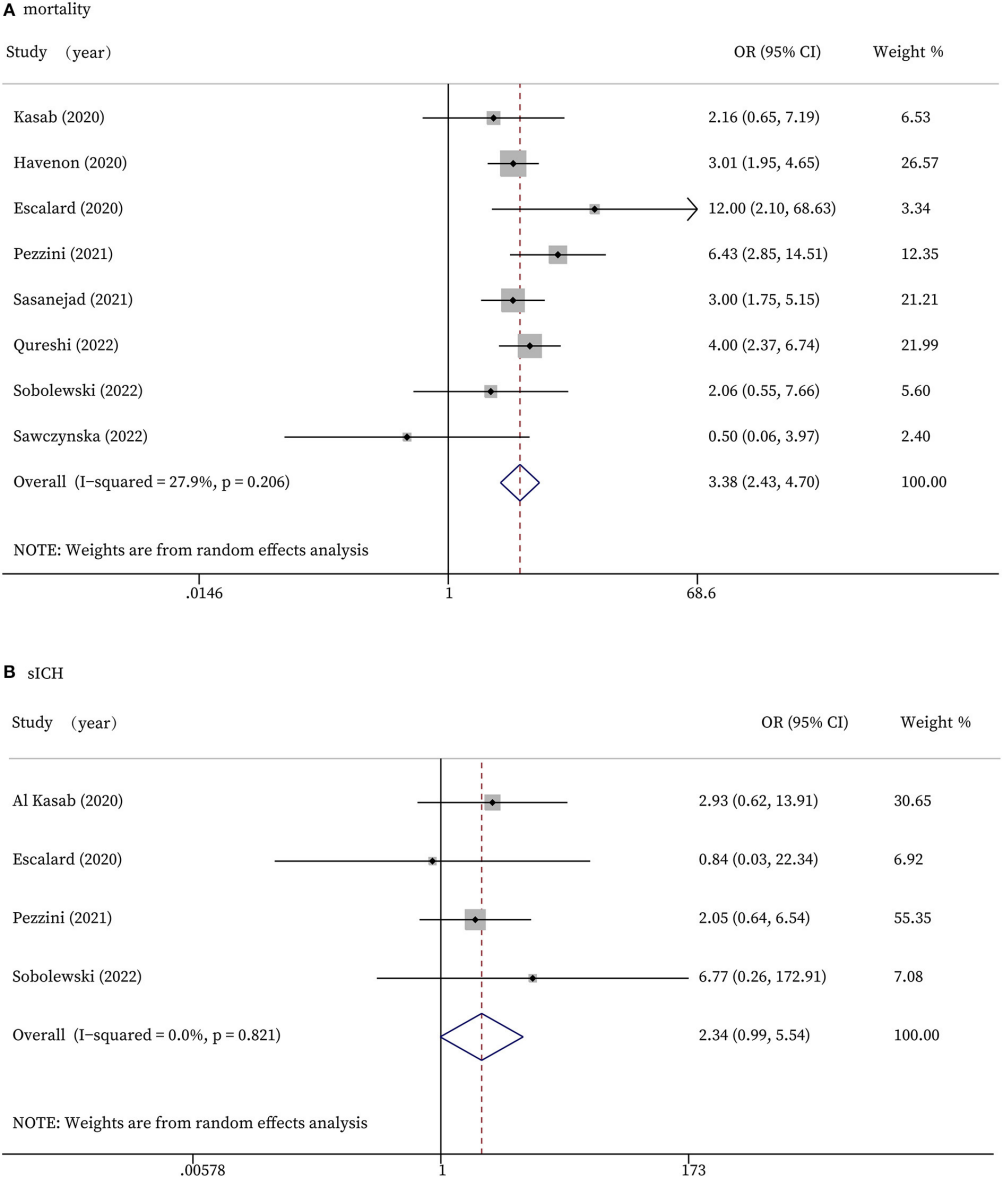
Forest plots for safety outcomes. **(A)**: In-hospital mortality; **(B)**: Symptomatic intracranial hemorrhage.

The safety outcomes include in-hospital mortality and sICH. As shown in Figure 3A, eight studies had the data of mortality. The forest plot indicated that stroke patients with COVID-19 infection who received recanalization treatments were associated with significantly higher mortality than those without COVID-19 infection (OR 3.38, 95% CI 2.43 to 4.70, *P* < 0.0001, *I*^2^ = 27.9%; Figure 3A). According to our meta-analyses, patients in COVID-19 positive group experienced more sICH than the control group, while the difference did not reach statistical significance (OR 2.34, 95% CI 0.99 to 5.54, *P* = 0.053, *I*^2^ = 0.0%; Figure 3B).

### Subgroup analyses

Subgroup analyses were implemented to assess the influence of median admission National Institutes of Health Stroke Scale (NIHSS) and different recanalization treatments on the outcomes. In studies that included patients with admission NIHSS < 15, patients without COVID-19 infection were associated with higher rates of functional independence on discharge (OR 0.38, 95% CI 0.20 to 0.72, *P* = 0.003; Table 2 and Supplementary Figure S4A) and successful recanalization (OR 0.36, 95% CI 0.16 to 0.80, *P* = 0.013; Table 2 and Supplementary Figure S4B) than patients with COVID-19 infection. In subgroup studies of different recanalization treatments on the outcomes, we found that in studies with patients with EVT treatment, patients with COVID-19 infection were associated with lower rates of functional independence on discharge (OR 0.27, 95% CI 0.12 to 0.60, *P* = 0.001; Table 2 and Supplementary Figure S6A) and longer length of hospital stay (WMD 6.57, 95% CI 1.71 to 11.43, *P* = 0.008; Table 2 and Supplementary Figure S6B). No significant difference was found between the time from stroke onset/door to treatment according to the subgroup analyses of different recanalization treatments or admission NIHSS (Table 2).

**Table 2:** Subgroup analysis of efficacy outcomes.

	**Efficacy outcomes**									
	**Functional independence on discharge**		**Successful recanalization**		**Length of hospital stay (days)**		**Time from stroke onset to treatment (min)**		**Time from door to treatment (min)**	
	**OR (95% CI)**	**P value**	**OR (95% CI)**	**P value**	**WMD (95% CI)**	**P value**	**WMD (95% CI)**	**P value**	**WMD (95% CI)**	**P value**
**1. Admission NIHSS**										
≥ 15	0.27 (0.07, 1.08)	0.065	0.67 (0.21, 2.19)	0.512	3.00 (−1.46, 7.46)	0.187^*^	−19.38 (−73.42, 34.66)	0.482	−26.00 (−71.81, 19.81)	0.266^*^
< 15	0.38 (0.20, 0.72)	0.003	0.36 (0.16, 0.80)	0.013	6.98 (−2.36, 16.32)	0.143	18.48 (−24.05, 61.01)	0.394	3.95 (−3.86, 11.75)	0.322
**2. Revascularization treatments**										
EVT	0.27 (0.12, 0.60)	0.001	N/A	N/A	6.57 (1.71, 11.43)	0.008	−6.46 (−86.56, 73.64)	0.874	6.86 (−14.45, 28.18)	0.528
IVT	0.44 (0.16, 1.26)	0.126^*^	N/A	N/A	1.81 (−2.57, 6.19)	0.419	4.89 (−18.99, 28.77)	0.688	0.86 (−4.71, 6.43)	0.762

CI, confidence interval; EVT, endovascular thrombectomy; IVT, intravenous thrombolysis; N/A, not applicable; NIHSS, National Institutes of Health Stroke Scale; OR, odds ratio; WMD, weighted mean difference.

^*^ Only one study was included.

In terms of mortality, significant difference was found in studies that included patients with admission NIHSS < 15 (OR 2.95, 95% CI 1.41 to 6.18, *P* = 0.004; Table 3 and Supplementary Figure S5A). In studies that included patients with different recanalization treatments, both EVT and IVT treatment subgroup indicated that patients with COVID-19 infection leaded to higher mortality (EVT: OR 3.46, 95% CI 1.76 to 6.82, *P* < 0.001; IVT: OR 3.34, 95% CI 2.33 to 4.80, *P* < 0.001; Table 3 and Supplementary Figure S7A). No significant difference of sICH was found according to the subgroup analyses of different recanalization treatments or admission NIHSS (Table 3). All the forest plots for subgroup analyses are available within the Supplemental material.

**Table 3:** Subgroup analysis of safety outcomes.

	**Safety outcomes**			
	**In-hospital mortality**		**sICH**	
	**OR (95% CI)**	**P value**	**OR (95% CI)**	**P value**
**1. Admission NIHSS**				
≥ 15	4.51 (0.85, 23.89)	0.077	2.33 (0.57, 9.51)	0.239
< 15	2.95 (1.41, 6.18)	0.004	2.35 (0.79, 6.99)	0.125
**2. Revascularization treatments**				
EVT	3.46 (1.76, 6.82)	<0.001	2.16 (0.88, 5.28)	0.092
IVT	3.34 (2.33, 4.80)	<0.001	6.77 (0.26, 172.91)	0.247^*^

CI, confidence interval; EVT, endovascular thrombectomy; IVT, intravenous thrombolysis; NIHSS, National Institutes of Health Stroke Scale; OR, odds ratio; sICH, symptomatic intracranial hemorrhage; WMD, weighted mean difference.

* Only one study was included.

### Risk of bias in included studies

The risk of bias in the ten studies based on the MINORS quality assessment was considered low, and none was excluded (Supplementary Table S3). The symmetrical funnel plot indicated no risk of publication bias (Supplementary Figure S8).

## Discussion

This study evaluated the efficacy and safety of recanalization therapy for AIS patients with COVID-19. We found that COVID-19 positive patients had significantly lower rates of functional independence at discharge, lower rates of successful recanalization, longer lengths of hospital stay, and higher mortality rates.

According to our study, the COVID-19 positive group who received recanalization therapy showed significantly lower rates of functional independence at discharge than COVID-19 negative group. Additionally, AIS patients with COVID-19 infection had a considerably longer length of hospital stay than those without COVID-19 infection. The previous report showed that ischemic stroke is more severe in patients with COVID-19, with a higher rate of patients who needed treatment in an ICU (31). In addition, patients with COVID-19 infection were more likely to have pneumonia, respiratory failure, acute kidney injury, septic shock, cardiac arrest, and require intubation or mechanical ventilation (26). These findings indicated that COVID-19 probably affected stroke patients through additional mechanisms. Therefore, these patients generally stayed in hospital for a longer period of time and have lower rates of functional independence at discharge. AIS patients with COVID-19 who were treated with EVT also had significantly lower rates of successful recanalization, as revealed by our study. COVID-19 may predispose patients to thrombosis, which is initially derived from the interaction of SARS-CoV-2 with ACE2; this will result in dysregulation of angiotensin signaling, subsequent inflammation, and tissue injury (32). This mechanism may also have a negative effect on the successful recanalization.

No significant difference between the two groups in the time from stroke onset to treatment was found. And our further subgroup analyses for the onset-to-needle time in AIS patients who received IVT and onset-to-groin puncture in those who received EVT still showed no significant difference between the two groups. Moreover, the time from door to treatment showed no significant difference. According to some studies, the time to treatment was significantly prolonged in COVID-19 positive patients with AIS (25, 28). Such a delay may be due to: the shortage of stroke team members, deceleration of evaluations, adherence to traffic restriction, and practice of preventive measures (33). However, in some research, the door-to-needle time was shortened through multidisciplinary collaboration and continuous process optimization despite the challenges posed by the COVID-19 pandemic (34). The study from Mathew et al. (9) also showed that the time from stroke onset to presentation to the hospital was reduced in stroke patients with COVID-19. This was an interesting observation that could be explained by less traffic on the roads due to the quarantine and the presence of other systemic symptoms that brought these patients to the hospital sooner. In addition, the time of enrollment of the patients may also be one of the important characteristics. As in the beginning of the pandemic, the in-hospital pathways were heavily affected, and preparation for the protective and specific treatment processes were inadequate, so time-to-treatment was frequently increased. As the stroke teams had time to adapt their processes, the previously identified delays were reduced. Studies that focused on comparisons between different COVID-19 periods with AIS patients infected by the SARS-CoV-2 virus in the same medical institution will further explain the phenomenon. Several studies including patients who were admitted with a principal diagnosis of AIS and received recanalization therapy reported that the time to treatment was similar in the COVID-19 positive group and COVID-19 negative group (19, 29). These different conditions in different areas or countries might explain that no significant difference was found between the time from stroke onset to treatment or the time from door to treatment. This might indicate that, as COVID-19 infection did not delay patients' recanalization treatment, the poorer outcomes of these patients might be due to the COVID-19 disease itself.

Regarding the safety outcomes of our meta-analyses, the mortality rate was significantly higher among patients who received recanalization therapy in the COVID-19 positive group than among those in the COVID-19 negative group, which was consistent with prior reports. Richer et al. found that the in-hospital mortality rate was significantly higher in patients with AIS and concurrent COVID-19 than in COVID-19 negative patients (35). Also, elevated d-dimer levels supported the increased risk of thromboembolism in COVID-19 patients and thus might increase their mortality rate (36). In addition, de Havenon et al. observed that more COVID-19 positive AIS patients were intubated, and had acute coronary syndrome, acute renal failure, and pulmonary emboli, which might also increase the mortality rate (37). The sICH is caused by an abnormally permeable blood-brain barrier resulting from ischemia of the capillary endothelium that allows for the leakage of blood cells (38). It is one of the severe complications of recanalization therapy in patients with AIS. According to our analysis, sICH occurred more frequently in COVID-19 positive group, while the difference did not reach statistical significance, the trend is clear. This may be due to the limitation of our data. As previously reported, the cytokine-driven imbalance in endogenous anticoagulant levels and hepatic dysfunction, especially in COVID patients, may contribute to coagulopathy with elevation in prothrombin time, and thrombocytopenia (18, 39). In addition to inflammation and coagulopathy, endothelial dysfunction with increased blood-brain barrier permeability after COVID-19 infection may also lead to more cases of sICH. A detailed assessment of coagulation to determine the risk: benefit ratio prior to recanalization therapy is necessary.

According to our subgroup analysis, we found that especially among studies including patients with admission NIHSS < 15, COVID 19 infection was associated with lower rates of functional independence, lower rates of successful recanalization, and higher in-hospital mortality rates. This might indicate that non-severe stroke patients who received recanalization treatments had poorer outcomes when infected by the SARS-CoV-2 virus.

This is the first meta-analysis to evaluate the efficacy and safety of recanalization therapy for AIS patients with COVID-19. Particular attention needs to be paid to the treatments for these patients, especially those non-severe stroke patients, to better improve their prognosis and decrease mortality. However, our study has some limitations. We acknowledge that the small sample size is a major limitation of the study. Most studies did not report the patients' long-term follow-up data. Also, the mechanisms of the poor outcomes of recanalization therapy for COVID-19 patients are not yet clear. We must admit that COVID-19 itself usually causes more severe strokes, and increases the risk of multisystemic complications, which might lead to poor outcomes in COVID-19 patients with AIS. In addition, whether COVID-19 causes AIS or whether it is a coincidental consequence of COVID-19 is difficult to determine, and the time from COVID-19 infection to AIS was not uniform across the included studies. Additionally, the results may change according to different variants, vaccinations and available COVID-19 treatments. Thus, further investigations are needed to better understand recanalization therapy for AIS patients with COVID-19.

## Conclusions

Our meta-analysis suggests that AIS patients with COVID-19 who received recanalization treatments had poorer outcomes than those without COVID-19. We need to pay more attention to the treatments for these patients to decrease mortality and morbidity. Further trials with long-term follow-up periods are necessary to evaluate the recanalization treatments for these AIS patients with COVID-19.

## Data availability statement

The original contributions presented in the study are included in the article/Supplementary material, further inquiries can be directed to the corresponding authors.

## Author contributions

ZiW and HT was the principal investigator and contributed in writing of the article. ZiW, HT, and ZC designed the study and developed the analysis plan. ZC, XW, and XY analyzed the data and performed meta-analysis. YQ and HC revised the manuscript and polish the language. ZC, ZhW, and GC supervised the project. All authors read and approved the final submitted paper.

## Funding

This work was supported by the Natural Science Foundation of Jiangsu Province (Grants No. BK20200203) and the National Natural Science Foundation of China (Grant No. 81873741).

## Conflict of interest

The authors declare that the research was conducted in the absence of any commercial or financial relationships that could be construed as a potential conflict of interest.

## Publisher's note

All claims expressed in this article are solely those of the authors and do not necessarily represent those of their affiliated organizations, or those of the publisher, the editors and the reviewers. Any product that may be evaluated in this article, or claim that may be made by its manufacturer, is not guaranteed or endorsed by the publisher.
