# Supplementary material for: Efficacy and safety of recanalization therapy for acute ischemic stroke with COVID-19: A systematic review and meta-analysis
Source: Front Neurol. 2022 Aug 30;13:984135. doi: 10.3389/fneur.2022.984135 (PMC9468325; doi:10.3389/fneur.2022.984135)
Supplement: Supplementary file 1 [file Data_Sheet_1.PDF]

SUPPLEMENTAL MATERIAL

1. Table S1: Search strategies and results

2. Table S2: Inclusion, exclusion criteria, and outcome assessments of the included studies

3. Table S3: Risk of bias based on MINORS quality assessment

4. Figure S1: sensitivity analysis of functional independence on discharge

5. Figure S2: sensitivity analysis of length of hospital stay

6. Figure S3: sensitivity analysis of time from stroke onset to treatment

7. Figure S4: Forest plots for subgroup analyses of admission NIHSS on efficacy outcomes

8. Figure S5: Forest plots for subgroup analyses of admission NIHSS on safety outcomes

9. Figure S6: Forest plots for subgroup analyses of recanalization treatments on efficacy outcomes

10. Figure S7: Forest plots for subgroup analyses of recanalization treatments on safety outcomes

11. Figure S8: Funnel plot assessing publication bias of odds ratio data reported in studies

Table S1: Search strategies and results

Database1 MEDLINE

("covid 19"[All Fields] OR "covid 19"[MeSH Terms] OR "covid 19 nucleic acid testing"[All Fields] OR "covid 19 nucleic acid testing"[MeSH Terms] OR "covid 19 serological testing"[All Fields] OR "covid 19 serological testing"[MeSH Terms] OR "covid 19 testing"[All Fields] OR "covid 19 testing"[MeSH Terms] OR "sars cov 2"[All Fields] OR "sars cov 2"[MeSH Terms] OR "severe acute respiratory syndrome coronavirus 2"[All Fields] OR "ncov"[All Fields] OR "2019 ncov"[All Fields] OR (("coronavirus"[MeSH Terms] OR "coronavirus"[All Fields] OR "cov"[All Fields]) AND 2019/11/01:3000/12/31[Date - Publication]) OR ("2019 ncov"[All Fields])) AND ("stroke"[MeSH Terms] OR "stroke"[All Fields] OR "strokes"[All Fields] OR "stroke s"[All Fields] OR ("cerebrovascular disorders"[MeSH Terms] OR ("cerebrovascular"[All Fields] AND "disorders"[All Fields]) OR "cerebrovascular disorders"[All Fields] OR ("cerebrovascular"[All Fields] AND "disease"[All Fields]) OR "cerebrovascular disease"[All Fields]) OR ("cerebral infarction"[MeSH Terms] OR ("cerebral"[All Fields] AND "infarction"[All Fields]) OR "cerebral infarction"[All Fields])) AND ("thrombolysis"[All Fields] OR ("thrombectomy"[MeSH Terms] OR "thrombectomy"[All Fields] OR "thrombectomies"[All Fields]) OR ("fibrinolytic agents"[Pharmacological Action] OR "fibrinolytic agents"[MeSH Terms] OR ("fibrinolytic"[All Fields] AND "agents"[All Fields]) OR "fibrinolytic agents"[All Fields] OR "thrombolytic"[All Fields] OR "thrombolytics"[All Fields]) OR ("revascularisation"[All Fields] OR "revascularisations"[All Fields] OR "revascularise"[All Fields] OR "revascularised"[All Fields] OR "revascularising"[All Fields] OR "revascularization"[All Fields] OR "revascularizations"[All Fields] OR "revascularize"[All Fields] OR "revascularized"[All Fields] OR "revascularizes"[All Fields] OR "revascularizing"[All Fields]) OR ("recanalisation"[All Fields] OR "recanalisations"[All Fields] OR "recanalise"[All Fields] OR "recanalised"[All Fields] OR "recanalising"[All Fields] OR "recanalization"[All Fields] OR "recanalizations"[All Fields] OR "recanalize"[All Fields] OR "recanalized"[All Fields] OR "recanalizers"[All Fields] OR "recanalizes"[All Fields] OR "recanalizing"[All Fields]))

Database2 EMBASE

#1 'covid 19'/exp

#2 'covid 19' OR (('covid'/exp OR covid) AND 19):ab,ti

#3 'sars cov 2':ab,ti

#4 '2019 ncov':ab,ti

#5 #1 OR #2 OR #3 OR #4

#6 'stroke'/exp

#7 'stroke':ab,ti

#8 'cerebrovascular disease':ab,ti

#9 'cerebral infarction':ab,ti

#10 #6 OR #7 OR #8 OR #9

#11 'thrombectomy'/exp

#12 'thrombolysis':ab,ti

#13 'thrombectomy':ab,ti

#14 'thrombolytic':ab,ti

#15 'revascularization':ab,ti

#16 'recanalization':ab,ti

#17 #11 OR #12 OR #13 OR #14 OR #15 OR #16

#18 #5 AND #10 AND #17

Database3 Cochrane

#1 MeSH descriptor: [COVID-19] explode all trees

#2 MeSH descriptor: [SARS-CoV-2] explode all trees

#3 (COVID 19):ti,ab,kw

#4 (SARS-CoV-2):ti,ab,kw

#5 (2019 nCoV):ti,ab,kw

#6 #1 OR #2 OR #3 OR #4 OR #5

#7 MeSH descriptor: [Stroke] explode all trees

#8 MeSH descriptor: [cerebrovascular disease] explode all trees

#9 MeSH descriptor: [Cerebral Infarction] explode all trees

#10 (stroke):ti,ab,kw

#11 (cerebrovascular disease):ti,ab,kw

#12 (cerebral infarction):ti,ab,kw

#13 #7 OR #8 OR #9 OR #10 OR #11 OR #12

#14 MeSH descriptor: [Thrombectomy] explode all trees

#15 (thrombolysis):ti,ab,kw

#16 (thrombectomy):ti,ab,kw

#17 (thrombolytic):ti,ab,kw

#18 (revascularization):ti,ab,kw

#19 (recanalization):ti,ab,kw

#20 #14 OR #15 OR #16 OR #17 OR #18 OR #19

#21 #6 AND #13 AND #20

Clinicaltrial.gov

- Condition or disease: (COVID-19 OR SARS-CoV-2 OR 2019-nCoV) AND (stroke OR cerebrovascular disease OR cerebral infarction)
- Other terms: thrombolysis OR thrombectomy OR thrombolytic OR revascularization OR recanalization

Table S2: Inclusion, exclusion criteria, and outcome assessments of the included studies

Studies	Inclusion Criteria	Exclusion Criteria	Efficacy Outcomes	Safety Outcomes
Al kasab[19] 2020	Consecutive patients who underwent MT for LVO during the peak 2 months of the COVID-19 (February-March or March-April 2020, depending on the individual site).	NA	Door to groin; Last known normal to groin；Number of passes during thrombectomy；Successful recanalization (mTICI ≥2 b)；Procedure duration； Door to reperfusion；Length of stay； Functional independence on discharge (mRS 0-2)；discharge mRS	In-hospital mortality; sICH
de Havenon[23] 2020	Patients whose date of hospital discharge was from April f1 to July 31 2020 and included those with ICD-10 codes for EVT and ischemic stroke.	Patients<18 years of age and those who were in a hospice at the time of admission were excluded.	Length of hospital stay; Length of intensive care unit stay; Favorable discharge	In-hospital mortality
Escalard[20] 2020	All consecutive patients with COVID-19 with AIS due to LVO treated in the institution during the 6 first weeks of the COVID-19 outbreak, between March 1st and April 15th, 2020, were included.	NA	Median time from onset to IVT; Median time from onset to arterial puncture; Median time from onset to recanalization; Median number of passes; First pass effect; Successful recanalization (TICI 2B-3); Median 24 h NIHSS; Dramatic early neurological improvement	In-hospital all-cause mortality; sICH
Pezzini[25] 2021	Patients with confirmed infection (COVID-19) and non-infected patients (non-COVID-19) who underwent reperfusion therapies for AIS between March 8 and April 30, 2020.	NA	Time from stroke onset to hospital admission; Time from stroke onset to brain imaging; Time from stroke onset to treatment; No recanalization; Stroke severity at 24 h, NIHSS score	In-hospital mortality; Any intracerebral bleeding; sICH
Sasanejad[27] 2021	Consecutive AIS patients treated with IV-tPA at seven stroke centers in Iran, one center in Athens, Greece and one in Dresden, Germany from February 18, 2019 to December 31, 2020.	Patients with unknown COVID-19 status were excluded from the study.	Door-to-needle time; NIHSS; Length of hospital stay	In-hospital mortality; Hemorrhagic transformation; sICH
Genchi[24] 2022	Prospective consecutive adult patients with COVID-19 and concomitant LVO-AIS treated with MT From February 2020 to March 2021, and with a cerebral thrombus available for histological analysis. Control group of COVID-19-negative patients from consecutive LVO-AIS patients admitted to the San Raffaele Hospital between July 2016 and November 2019, treated by MT and with a thrombus available for analysis.	NA	Onset to groin time; successful recanalization (mTICI ≥2 b); 90-day mRS > 2	NA

Qureshi[26] 2022	The data from the Cerner de-identified COVID-19 dataset which included data from 62 contributing Cerner Real-World Data health systems from United States between December 1, 2019 and January 1, 2021. The ICD-10-CM primary diagnosis codes I63, I65 and I66 were used to identify the patients admitted with acute ischemic stroke.	Patients in whom no previous medical encounter occurred during the past 5 years were excluded. Encounters with missing data for certain nonessential variables such as gender were included.	NA	In-hospital mortality
Sobolewski[29] 2022	Study population consisted of patients who were treated only with IVT between 15 September 2020 and 30 November 2020. For SARS-CoV-2-infected group, patients with positive test performed within 3 days of admission were included.	NA	mRS 0-2 at discharge; Length of stay in hospital	In-hospital mortality; sICH
Jabbour[30] 2022	Consecutively admitted patients with COVID-19 with concomitant acute LVO across 50 comprehensive stroke centers. The control group constituted historical controls of patients presenting with LVO and receiving a MT between January 2018 and December 2020.	NA	successful recanalization (mTICI 3); functional outcome at discharge	NA
Sawczyńska[28] 2022	The medical documentation of patients who had undergone MT for AIS in the Comprehensive Stroke Centre (CSC) of the University Hospital in Krakow, Poland during the COVID-19 pandemic between March 2020 and May 2021 were analyzed. Patients with a COVID-19 infection confirmed at admission or in the referring hospital, or before hospitalization were included. The control group consisted of AIS patients treated with MT in the CSC between March 2020 and February 2021, who tested negative for SARS-CoV2 at admission.	Patients who were negative for COVID-19 at admission but who tested positive during hospitalization, or those who were transferred to another center and therefore lost to follow-up.	Time from stroke onset to admission; Time from admission to groin puncture; Full reperfusion (TICI 2b-3); NIHSS at discharge; mRS at discharge; Days of hospitalization	In-hospital mortality

AIS: acute ischemic stroke; **EVT:** endovascular thrombectomy; **IVT:** Intravenous thrombolysis; **LVO:** large vessel occlusion; **MT:** mechanical thrombectomy; **mRS:** modified Rankin Scale; **NIHSS**: National Institutes of Health Stroke Scale; **sICH**: symptomatic intracerebral hematoma; **TICI:** Treatment in Cerebral Infarction; IC**D-10**: International Classification of Diseases, Tenth Revision.

Table S3 Risk of bias based on MINORS quality assessment

NO	item	Al Kasab[19] 2020	de Havenon[23] 2020	Escalard[20] 2020	Pezzini[25] 2021	Sasanejad[27] 2021	Genchi[24] 2022	Qureshi[26] 2022	Sobolewski[29] 2022	Jabbour[30] 2022	Sawczyńska[28] 2022
1	A clearly stated aim	2	2	2	2	2	2	2	2	2	2
2	Inclusion of consecutive patients	2	2	2	2	2	2	2	2	2	2
3	Prospective collection of data	2	1	2	2	2	2	0	1	1	1
4	Endpoints appropriate to the aim of the study	2	2	2	2	2	2	2	2	2	2
5	Unbiased assessment of the study endpoint	1	2	1	1	1	1	2	1	2	1
6	Follow-up period appropriate to the aim of the study	2	2	2	2	2	2	2	2	2	2
7	Loss to follow up less than 5%	2	2	2	2	2	2	2	2	2	2
8	Prospective calculation of the study size	0	0	0	0	0	0	0	0	0	0
9	An adequate control group	2	2	2	2	2	2	2	2	2	2
10	Contemporary groups	2	2	2	2	2	1	2	2	1	2
11	Baseline equivalence of groups	2	1	2	2	2	2	2	1	1	2
12	Adequate statistical analyses	2	2	1	2	2	2	2	2	2	2
13	Total score	21	20	20	21	21	20	20	19	19	20

The items are scored 0 (not reported), 1 (reported but inadequate) or 2 (reported and adequate). The global ideal score being 16 for non-comparative studies and 24 for comparative studies.

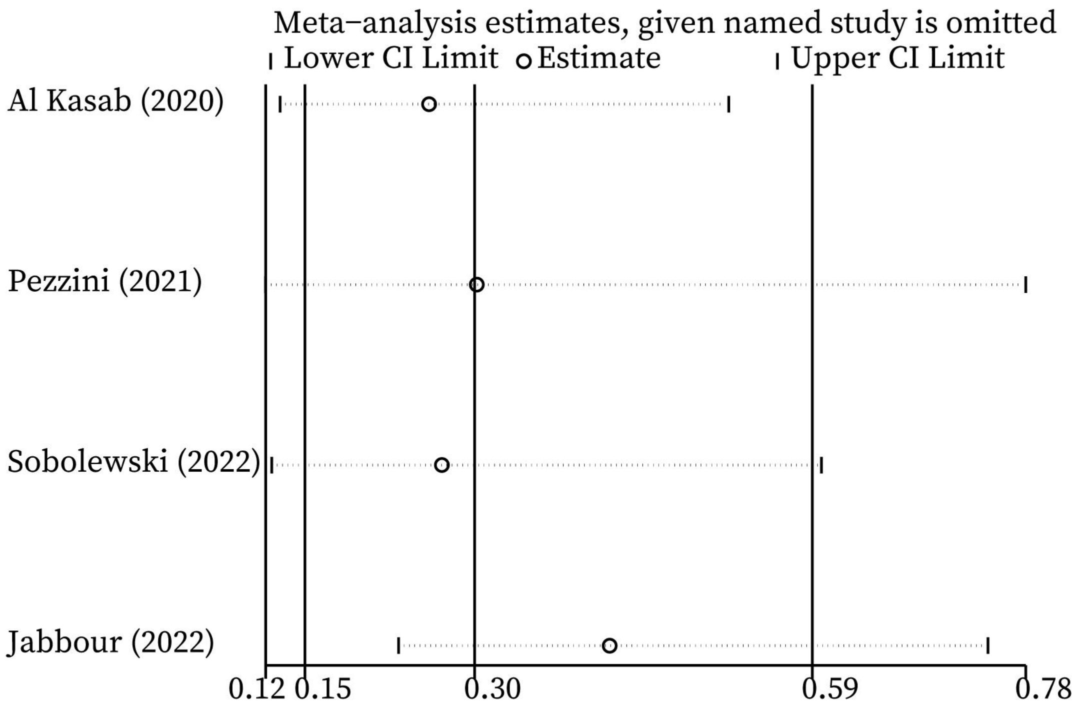

Figure S1. sensitivity analysis of functional independence on discharge

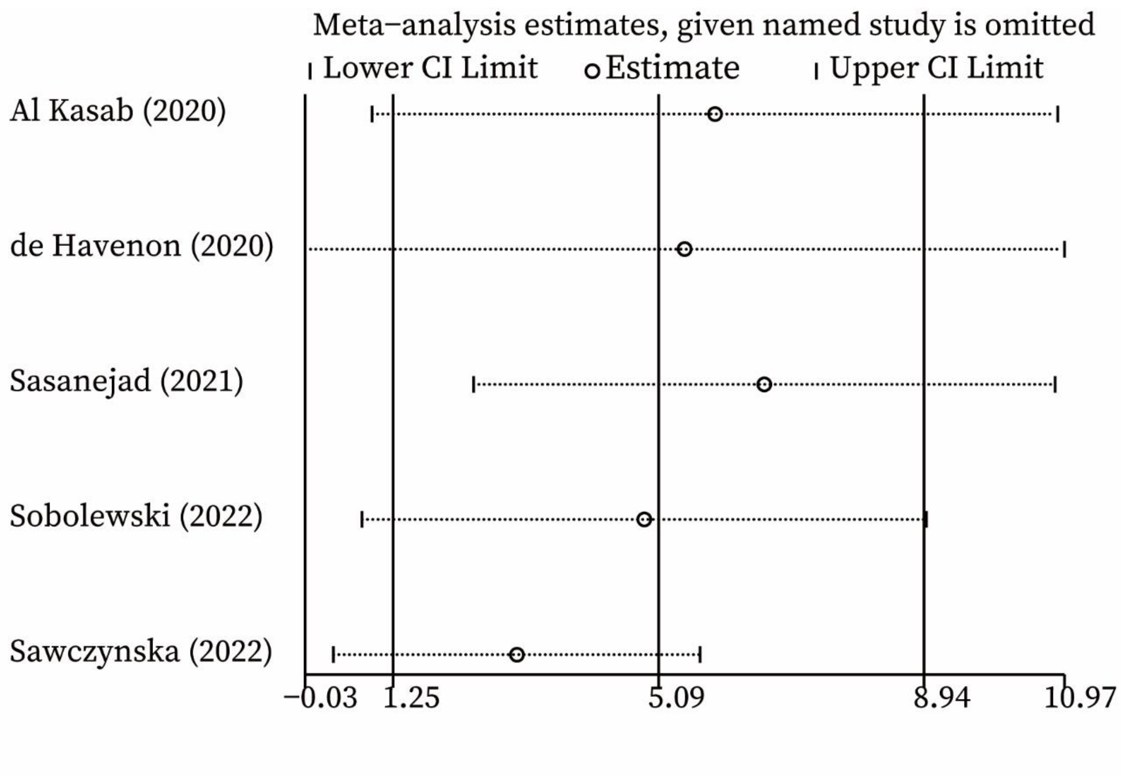

Figure S2. sensitivity analysis of length of hospital stay

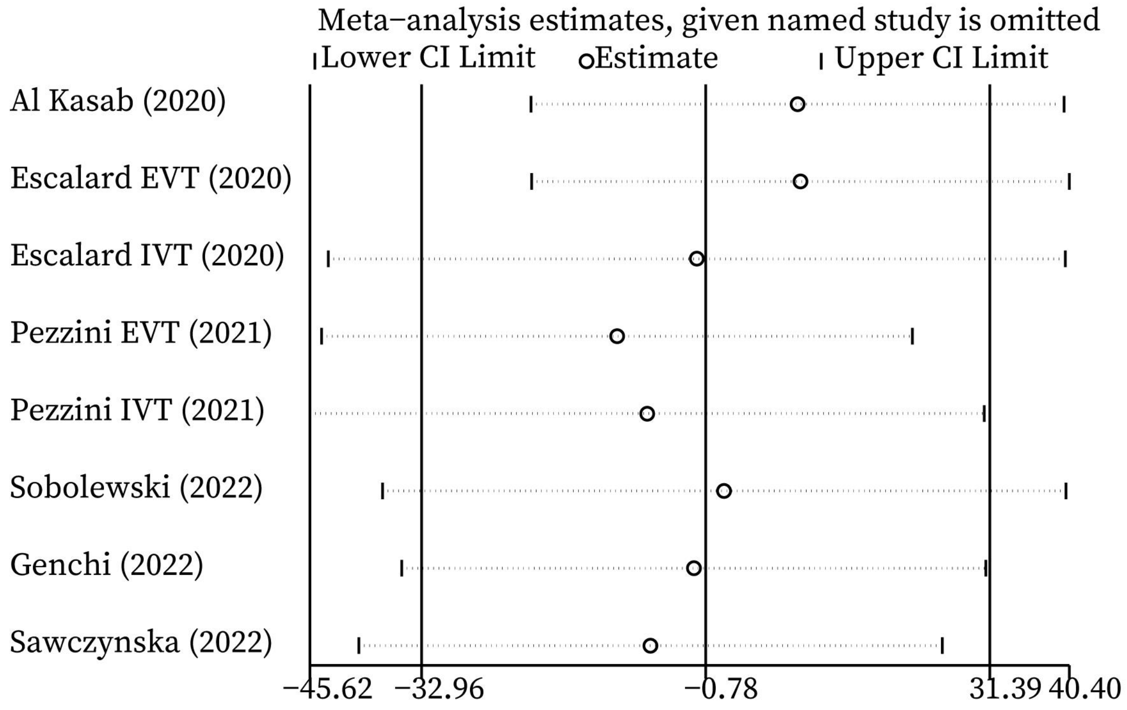

Figure S3. sensitivity analysis of time from stroke onset to treatment

A. Functional independence on discharge

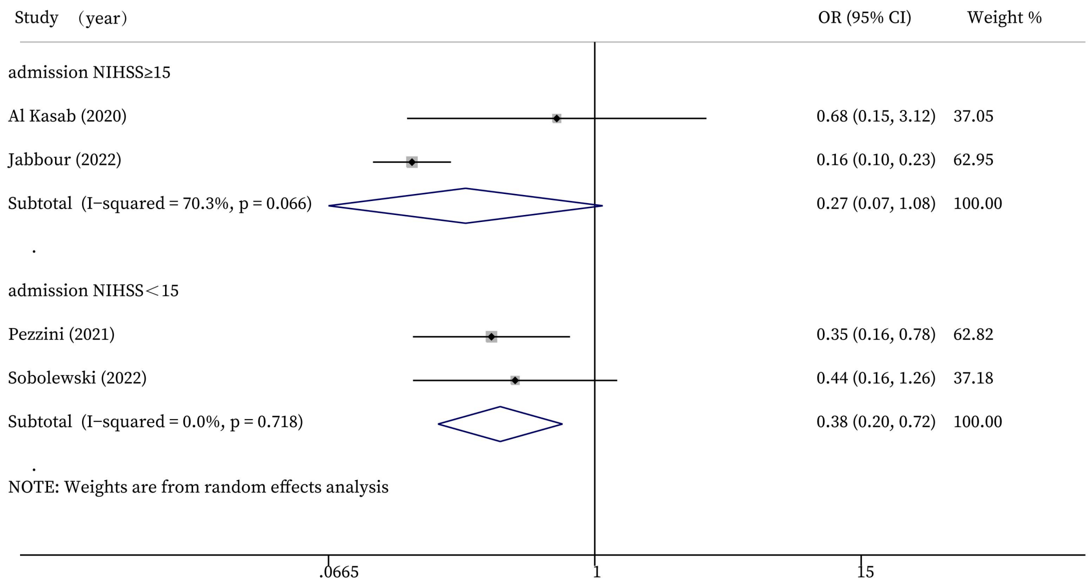

B. successful recanalization

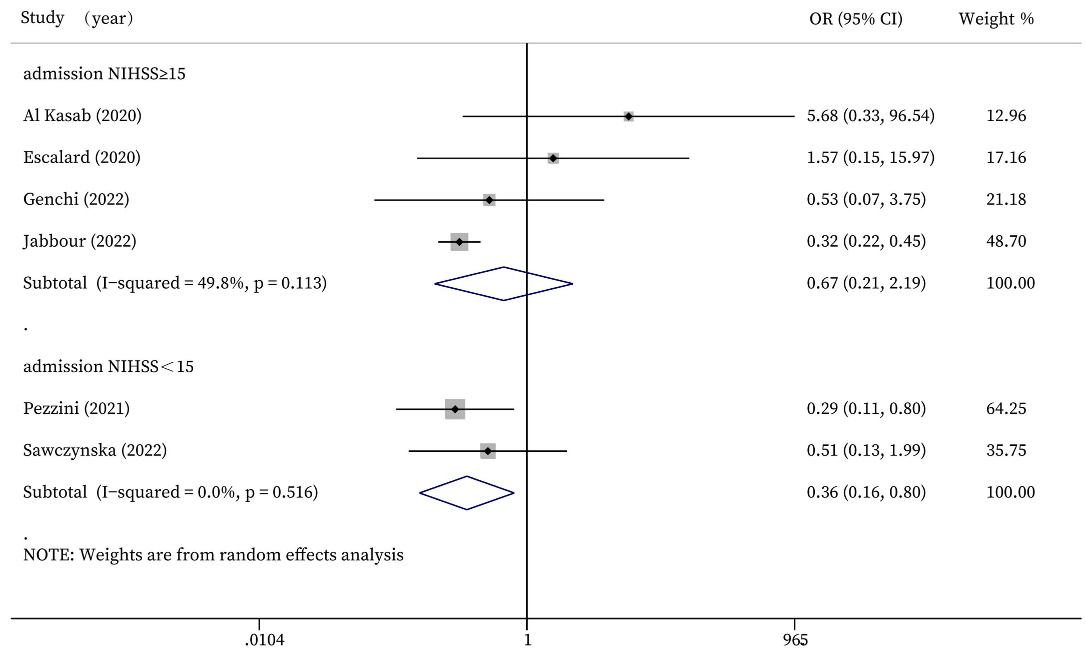

C. length of hospital stay (days)

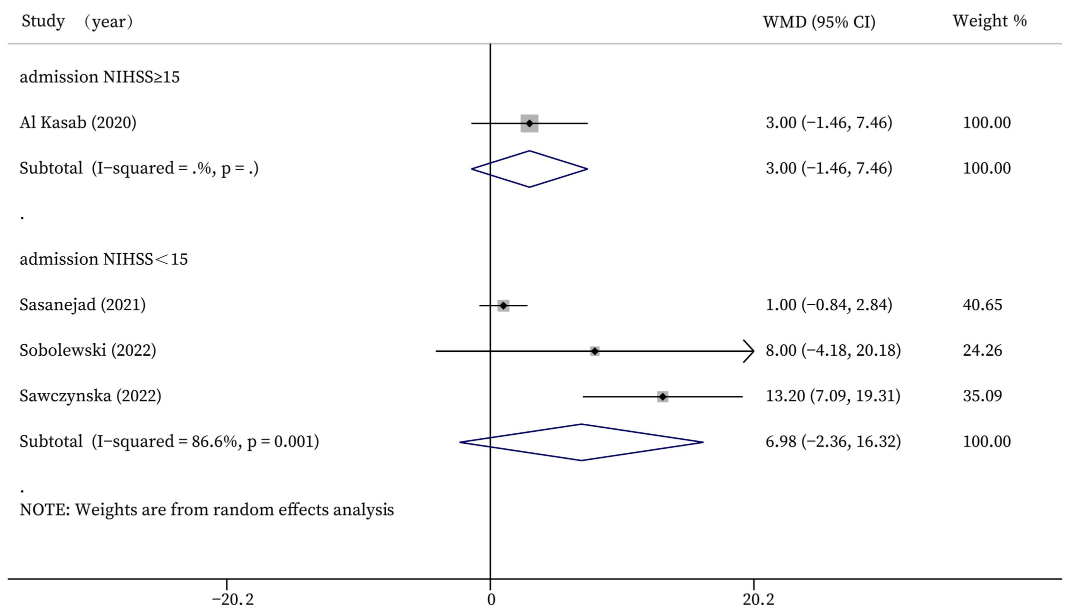

D.time (min) from stroke onset to treatment

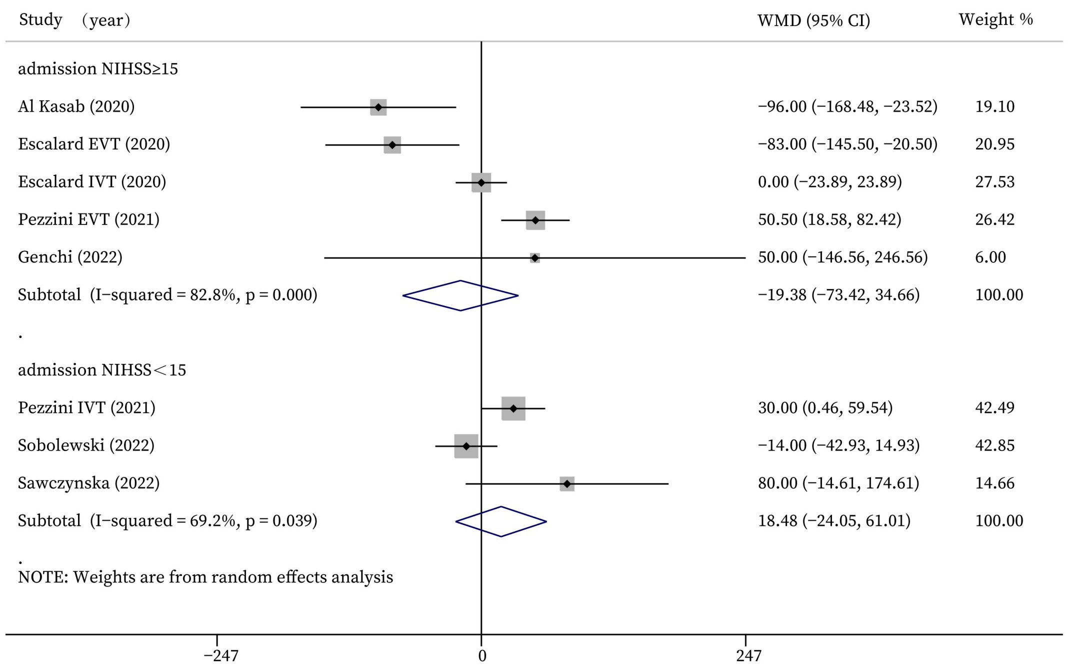

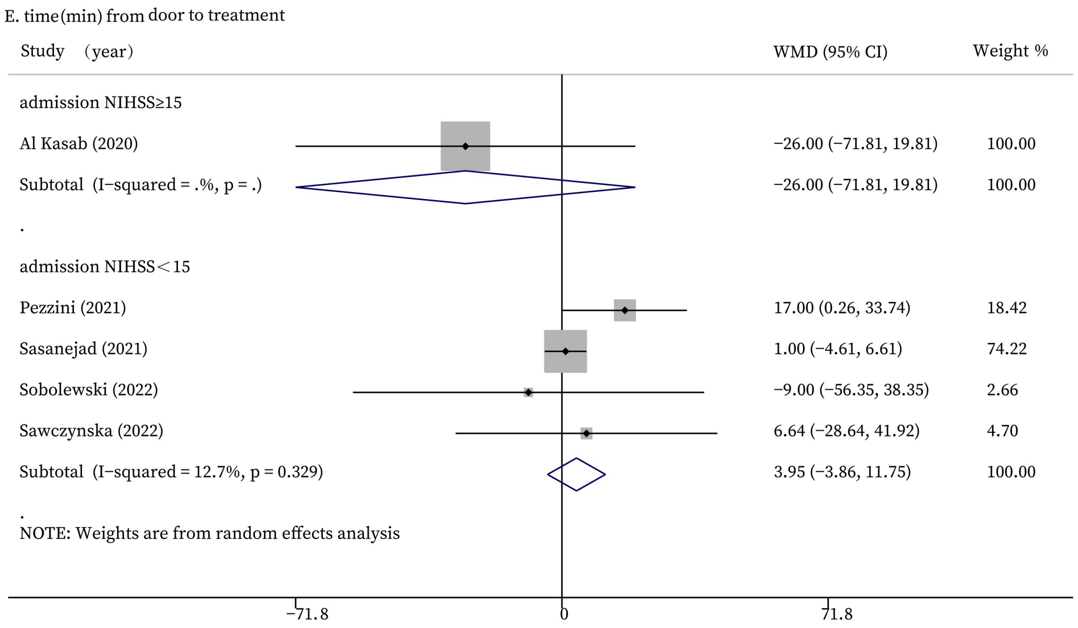

Figure S4. Forest plots for subgroup analyses of admission NIHSS on efficacy outcomes. A: Functional independence on discharge; B: successful recanalization; C: length of hospital stay (days); D: time (min) from stroke onset to treatment; E: time (min) from door to treatment.

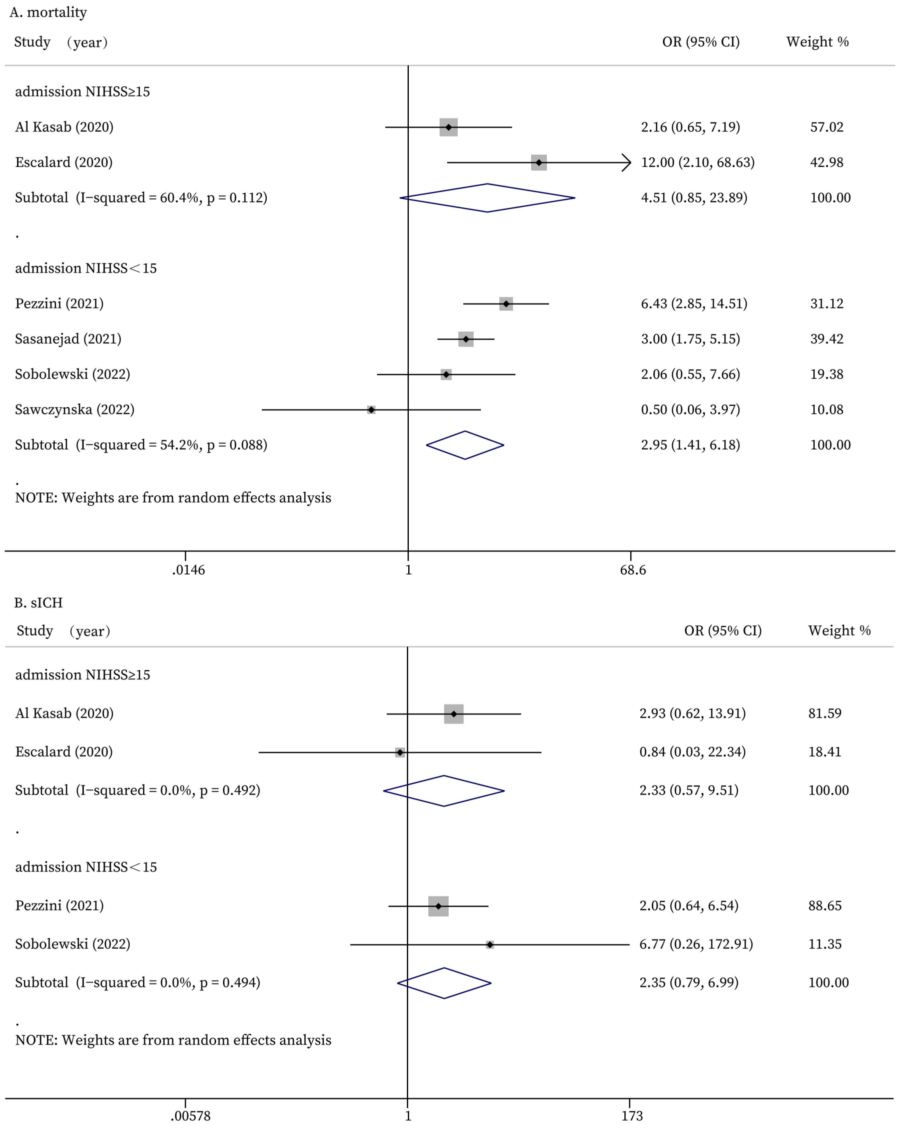

Figure S5. Forest plots for subgroup analyses of admission NIHSS on safety outcomes. A: mortality; B: symptomatic intracranial hemorrhage.

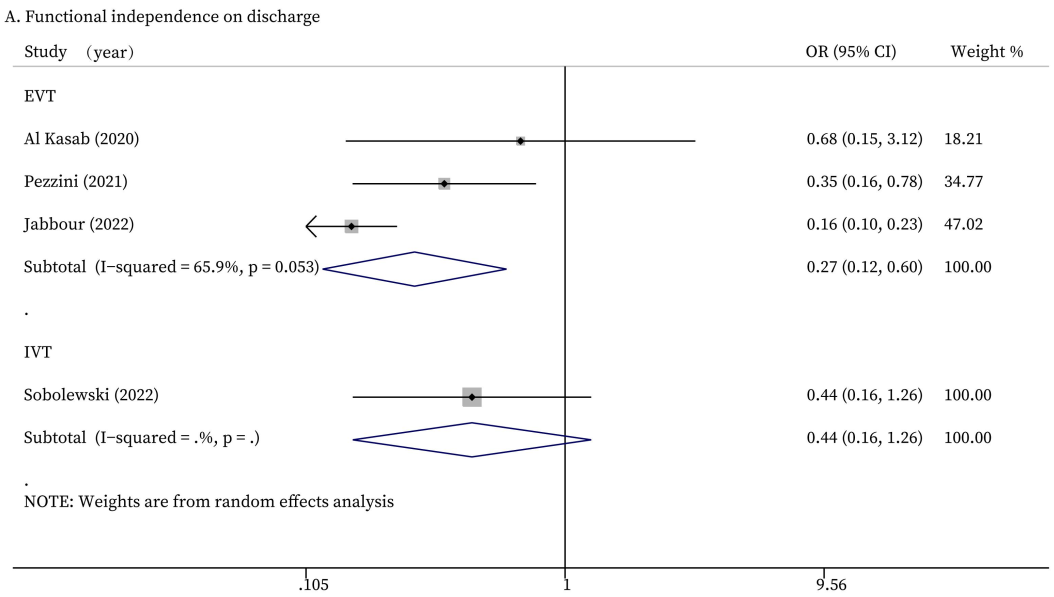

A. Functional independence on discharge
Study (year)
OR (95% CI)
Weight %
EVT
Al Kasab (2020)
0.68 (0.15, 3.12)
18.21
Pezzini (2021)
0.35 (0.16, 0.78)
34.77
Jabbour (2022)
0.16 (0.10, 0.23)
47.02
Subtotal (I−squared = 65.9%, p = 0.053)
0.27 (0.12, 0.60)
100.00
IVT
Sobolewski (2022)
0.44 (0.16, 1.26)
100.00
Subtotal (I−squared = .%, p = .)
0.44 (0.16, 1.26)
100.00
NOTE: Weights are from random effects analysis
.105
1
9.56

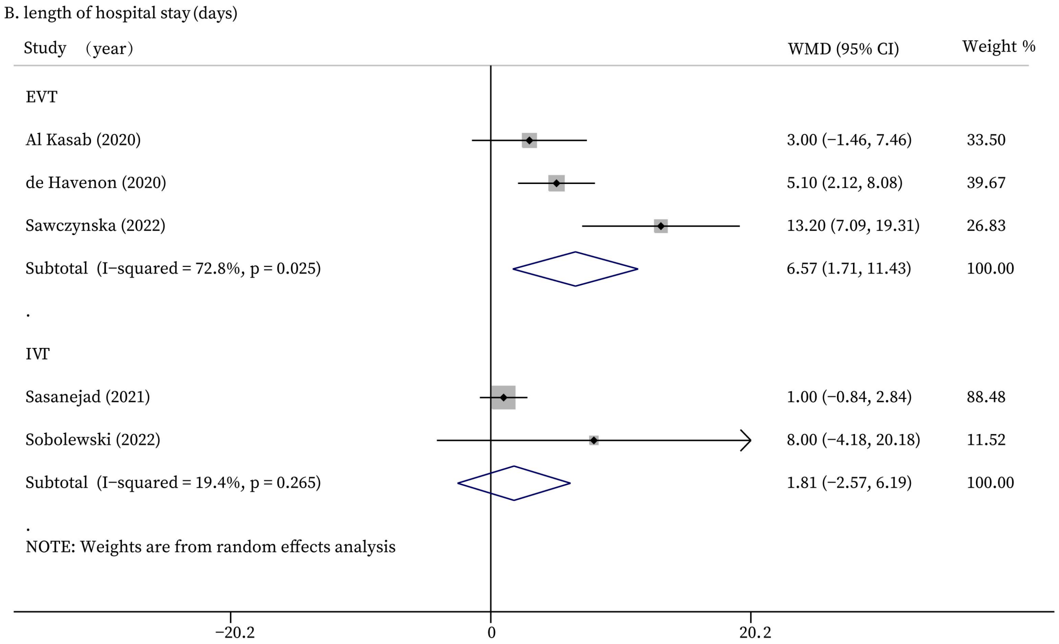

B. length of hospital stay (days)
Study (year)
WMD (95% CI)
Weight %
EVT
Al Kasab (2020)
3.00 (−1.46, 7.46)
33.50
de Havenon (2020)
5.10 (2.12, 8.08)
39.67
Sawczynska (2022)
13.20 (7.09, 19.31)
26.83
Subtotal (I−squared = 72.8%, p = 0.025)
6.57 (1.71, 11.43)
100.00
IVT
Sasanejad (2021)
1.00 (−0.84, 2.84)
88.48
Sobolewski (2022)
8.00 (−4.18, 20.18)
11.52
Subtotal (I−squared = 19.4%, p = 0.265)
1.81 (−2.57, 6.19)
100.00
NOTE: Weights are from random effects analysis
−20.2
0
20.2

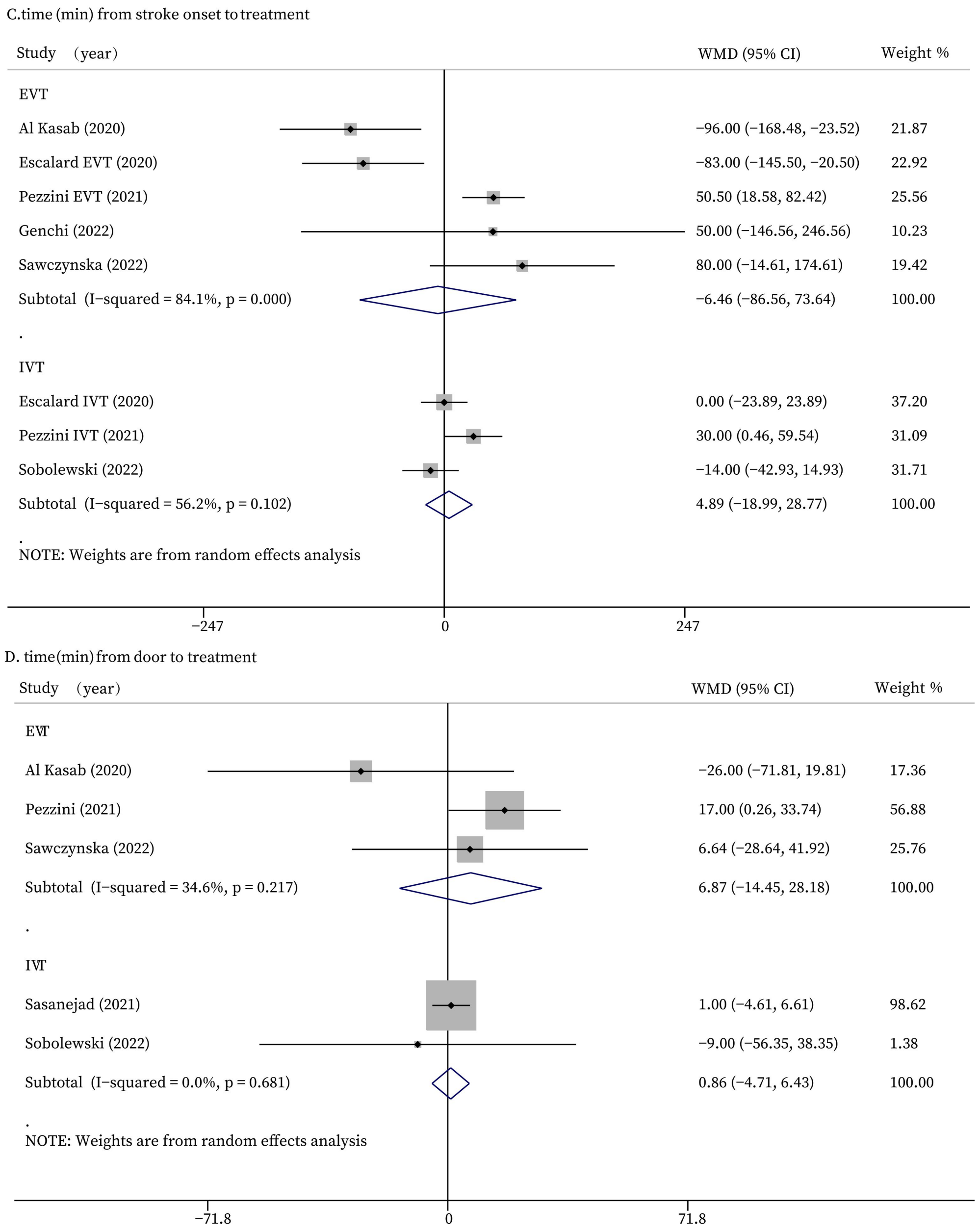

Figure S6. Forest plots for subgroup analyses of recanalization treatments on efficacy outcomes. A: Functional independence on discharge; B: length of hospital stay (days); C: time (min) from stroke onset to treatment; D: time (min) from door to treatment.

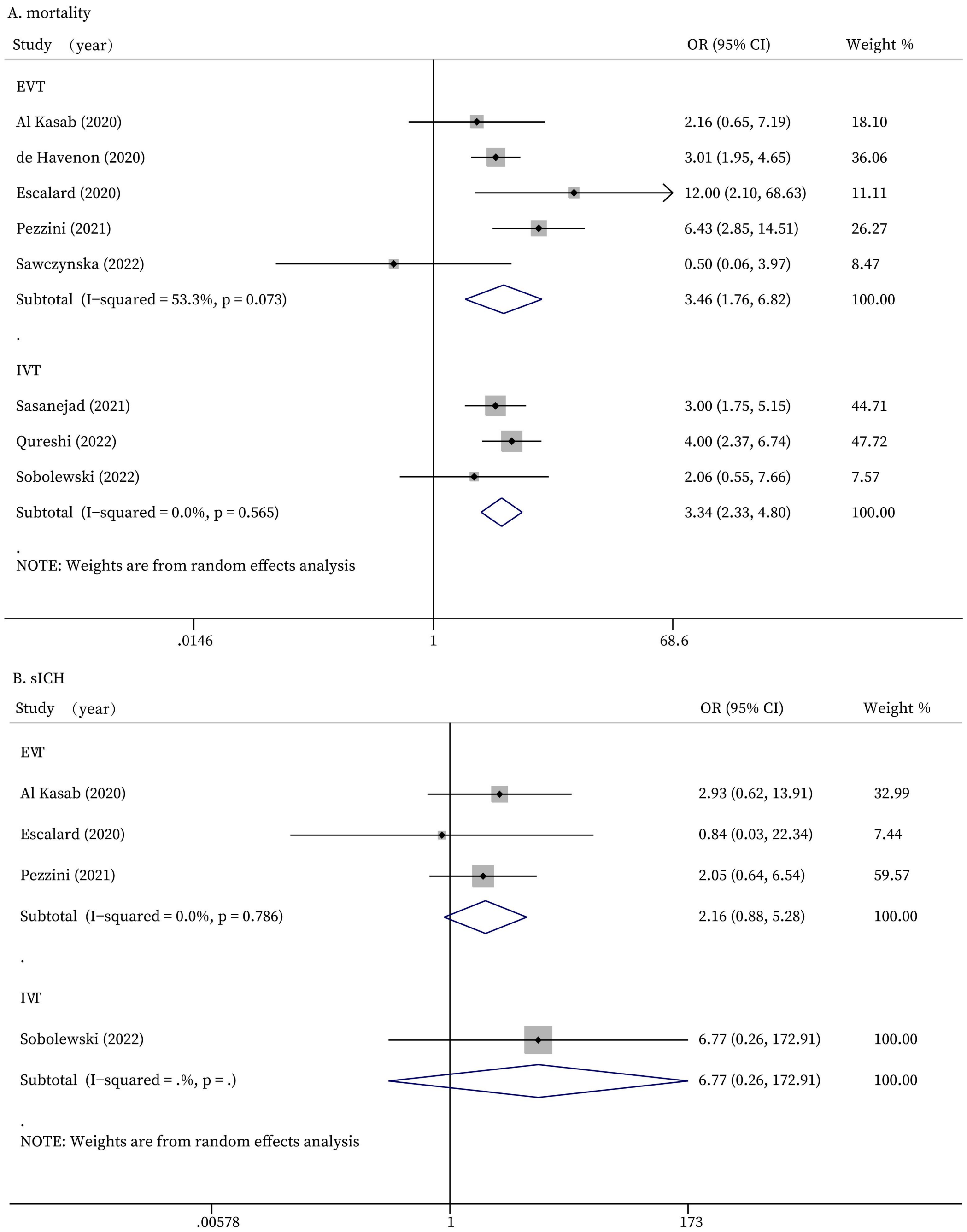

Figure S7. Forest plots for subgroup analyses of recanalization treatments on safety outcomes. A: mortality; B: symptomatic intracranial hemorrhage.

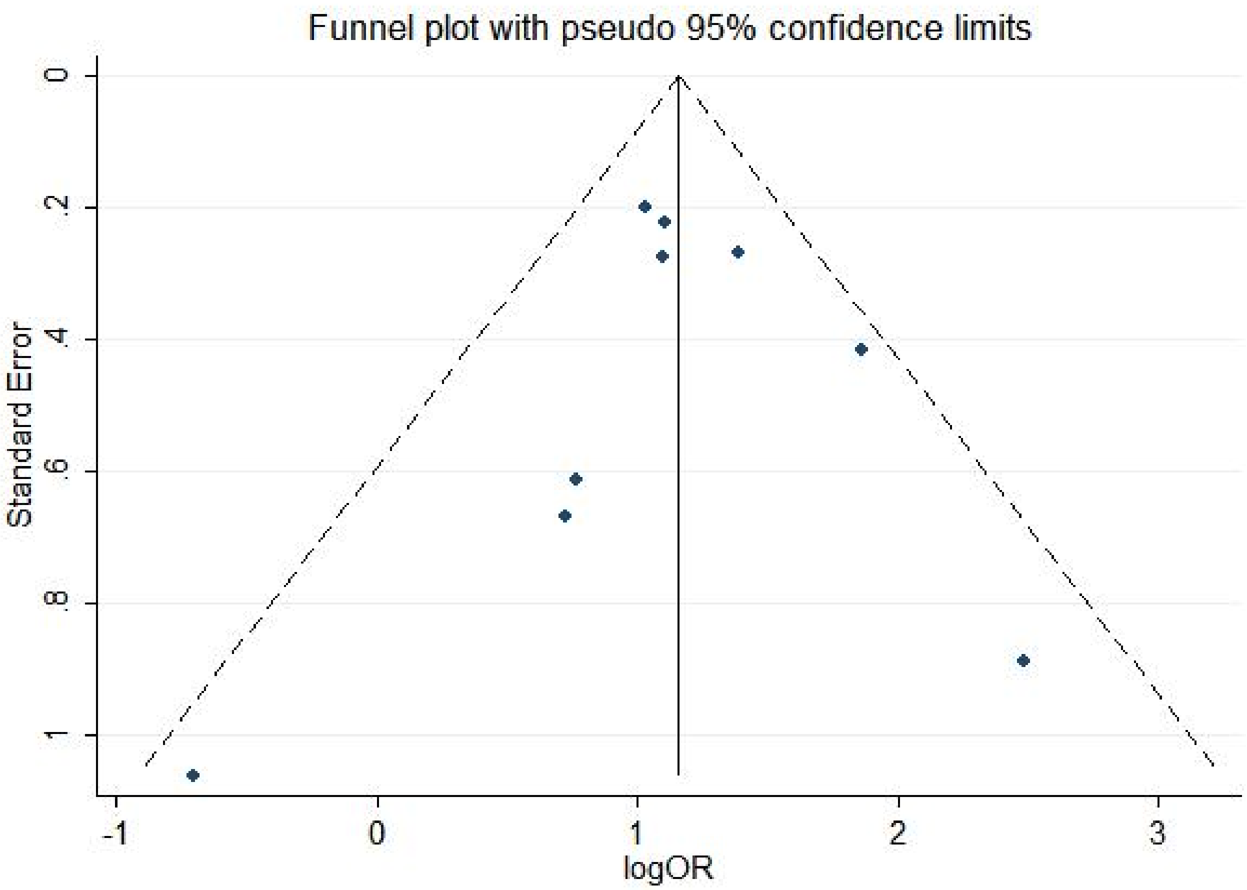

Figure S8: Funnel plot assessing publication bias of odds ratio data reported in studies
